# A novel hantavirus identified in bats (*Carollia perspicillata*) in Brazil

**DOI:** 10.1038/s41598-024-56808-6

**Published:** 2024-03-15

**Authors:** Mike Barbosa dos Santos, Nádia Koide Albuquerque, Sandro Patroca da Silva, Fábio Silva da Silva, Daniel Damous Dias, Samira Brito Mendes, Taciana Fernandes Souza Barbosa Coelho, Maria Claudene Barros, Ana Cecília Ribeiro Cruz

**Affiliations:** 1 Arbovirology and Hemorragic Fever Department, Evandro Chagas Institute, Ananindeua, Pará 67030-000 Brazil; 2 Federal University of Pará, Institute of Biologic Science, Belém, Pará 66075-750 Brazil; 3 Laboratory of Genetics and Molecular Biology, State University of Maranhão, Caxias, Maranhão 65604-380 Brazil; 4 Laboratory of Genetics and Molecular Biology, State University of Maranhão, São Luís, Maranhão 650-8805 Brazil

**Keywords:** Virology, Metagenomics

## Abstract

Bats play an essential role in maintaining ecosystems. Their unique characteristics increase the likelihood of interactions with various species, making them a potential source for the emergence and spread of infectious diseases. Hantaviruses are continuously expanding their range of hosts. This study presents the identification of a partial genome associated with *Hantavirus* in samples collected from neotropical bats. We conducted a metagenomic study using samples from *Carollia perspicillata* in Maranhão, Brazil. Tissue fragments were used for RNA extraction and subsequent sequencing. The resulting data was subjected to bioinformatic analysis. A sequence showing an identity of 72.86% with the L gene in the reference genome was obtained. The phylogenetic analysis revealed the study sequence, denoted as *Buritiense*, clustering within the *Mobatvirus* clade. The intragroup analysis showed a broader dispersion and were markedly asymmetric. This observation suggests the possibility that *Buritiense* could potentially represent a new species within the bat-borne hantaviruses, but further analyses are needed to provide additional insights if bats plays a role as reservoirs and the potential for transmission to human populations.

## Introduction

Bats play an essential role within ecosystems, such as insect population control and plant pollination. The adaptation of bats to a variety of environments and their flying capabilities afford them the opportunity to come into contact with various species, including rodents, birds, and other mammals, rendering them a potential source for the emergence and dissemination of infectious diseases. Their immunological and physiological characteristics of bats may enable them to harbor viruses in a manner that does not induce illnesses within themselves, but these viruses could potentially be transmitted^1,2^.

Hantaviruses are one of the various types of emerging viruses that have presented serious public health issues in the last decades. In humans, *Hantavirus* (HV) infections can lead to two significant clinical syndromes, Hemorrhagic Fever with Renal Syndrome (HFRS) and *Hantavirus* Cardiopulmonary Syndrome (HCPS). HFRS, characterized by fever, hemorrhage, and renal complications, is primarily found in Asian countries. On the other hand, HCPS is more prevalent in the Americas, manifests with severe respiratory and cardiovascular symptoms, often leading to a critical condition^3^. Recent cases of hantaviruses have been confirmed in South America, in countries such as Argentina, Panama, and Brazil^4^.

The first description of HV emerged from studies on the origin of hemorrhagic fever in over 3000 soldiers during the Korean conflict (1950–1953). However, the causative agent of the disease remained unknown until the early 1980s when the *Hantaan virus* was isolated, found in the lungs of the striped field mouse (*Apodemus agrarius*), its natural reservoir^3,5^.

As the costs of *Next-Generation Sequencing* (NGS) have declined, obtaining complete viral sequences or draft genomes has become more attainable without the need for cell culture. The characterization of the virome in various host species now serves as a valuable tool for zoonotic surveillance. The metagenomic approach not only facilitates the detection of well-known viruses of significance, but also opens the door to the identification of novel viral species^6^. HV are primarily transmitted by rodents, but bats in Brazil have also been identified as hosts for different HV species^7–9^.

Members of the family *Hantaviridae* are enveloped, spherical with a diameter from 80 to 120 nm and have segmented negative-stranded RNA linear genome. The genome encodes 3 segments L, M and S. The segment L encodes RNA-dependent RNA polymerase (RdRp), the M segment encodes the glycoprotein precursor (GPC) which is later cleaved into G1 and G2 and the S segment encodes the nucleoprotein (NP). These proteins are essential in the virus life cycle, replication, and interactions with host cells. Among the four subfamilies, *Mammahantavirinae* have a particular significance, as it includes important genera, such as *Orthohantavirus* and *Mobatvirus*, which are known to be hosted by rodents and bats respectively^3,10^.

The increasing number of highly impact viral diseases in public health is directly linked to environmental and climatic changes. These impacts contribute to the spread of zoonotic diseases, altering the endemic habitats of wildlife, leading to changes in species distribution, as well as a reduction in the barrier between wildlife and the human population. This, in turn, disrupts the ecosystem and exposes humans to emerging pathogens. In the Brazilian forest, this scenario is particularly concerning, given the incidences of deforestation and disturbance of the natural fauna^11,12^.

HV is among the viruses extending its connections to new hosts, having also been identified in domestic animals. Environmental and economic factors are intensifying human interaction with the virus hosts, giving rise to scenarios where new strains with potentially heightened infectivity and pathogenicity can emerge^12,13^. For this reason, we may be facing new challenges in disease prevention and control, as we understand that the HV type is intimately linked to host selection and regional adaptation, continually evolving through genetic recombination^13,14^. This study reports the discovery of a partial genome related to HV in samples obtained from neotropical bats captured in the Brazilian biome.

## Results

After bioinformatic analysis, a contig of 6291 nucleotides (nt) has been recovered, which encodes a protein comprising 2096 amino acids (aa). The 5’ and 3’ ends are missing in this genome. The BlastX results showed 99% query coverage and 73.21% identity with *Dakrong virus* (GenBankID: MG663536), corresponding to segment L, which encodes RNA polymerase. This novel sequence has been named *Buritiense virus*.

**Figure 1:**
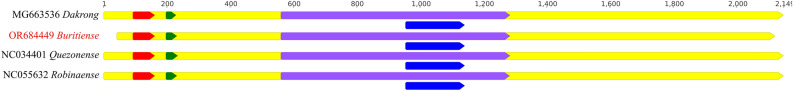
Conserved domains of Mobatvirus. Demonstration of the different functional domains of proteins from various viruses belonging to the genus *Mobatvirus*. The yellow arrow corresponds to the coding region of the proteins. The different domains are shown, such as RNA endonuclease, cap-snatching (TIGR04202) in red, RNA-dependent RNA polymerase (PF12426) in green, *Bunyavirus* RNA-dependent RNA polymerase (PF04196) in purple, and RdRp of negative ssRNA viruses with segmented genomes catalytic domain profile (PS50525) in blue.

The sequences were translated and aligned to facilitate phylogenetic inference analysis based on the partial L segment of HV, encompassing positions between sites 137–6398, related to the RdRp domain Fig. 1.

The nt and aa identity matrices were consolidated in Table S1. At *Mobatvirus* genus, the aa matrix showed a reduced genetic distance between the recently sequenced *Buritiense* compared to the *Dakrong* and *Robinaense* HV species, with values of 0.2672 and 0.2676, respectively. When assessed at the nt level, the distances were 0.4214 and 0.4369 in comparison to *Dakrong* and *Robinaense*, respectively.

On the other hand, at *Orthohantavirus* genus, the species *Boweense* and *Jejuense* exhibited higher genetic distances in relation to *Buritiense*, with values of 0.3651 and 0.3642 for the aa matrix and 0.5443 and 0.5290 for the nt matrix. The aa tree was used to represent the phylogeny.

**Figure 2:**
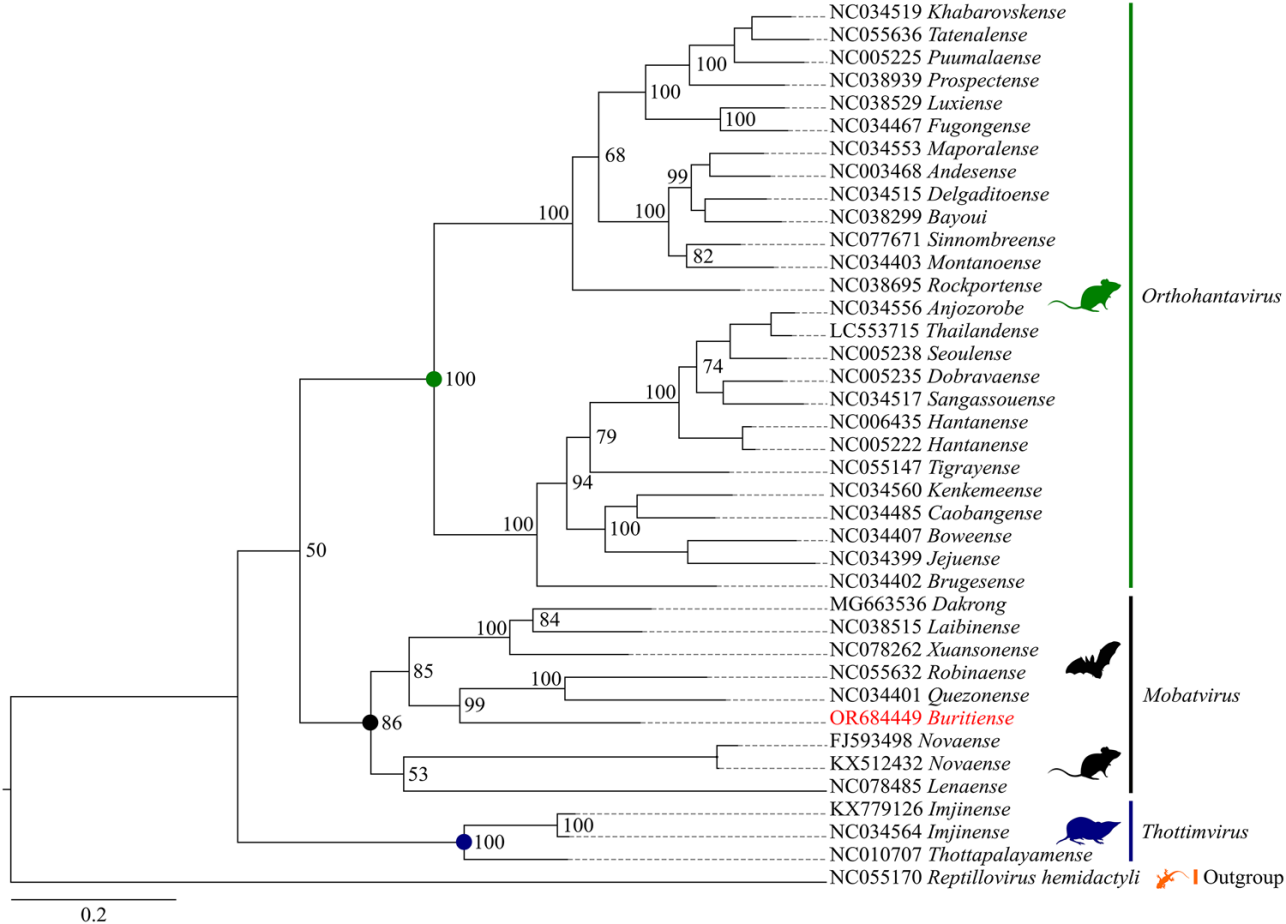
Phylogenetic tree. Reconstruction of the phylogeny of *Hantaviridae* family representative viruses was performed using the Maximum Likelihood method. The bootstraping values defined for a run of 1.000 repetitions are displayed on each node. Colored circles indicate the clades of the main taxonomic groups, with the newly sequenced taxon highlighted in red. The study sequence, *Buritiense*, forms a distinct branch within bats clade, near to *Quenzonense* and *Robinaense*.

Assessment of the phylogenetic signal in the sequence set used to reconstruct the *Hantaviridae* phylogeny revealed that 98.7% of quartets were resolved (see Supplementary Fig. S1). In the phylogenetic inference, three HV genera are observed, with the *Buritiense* strain grouping within the *Mobatvirus* clade, which in turn exhibited two distinct subclades: one for bat hosts and another for rodents with a support of 85%. The *Buritiense* strain formed an isolated branch of clade within the bat subclade supported at 99%, closely related to the *Quenzonense* and *Robinaense* species as illustrated in Fig. 2.

Two boxplot graphs were generated to illustrate the intergroup and intragroup genetic divergence for the genera *Orthohantavirus*, *Mobatvirus*, and *Thottimvirus*. On the graph A, the bat subclade *Mobatvirus* I intergroup demonstrates a narrow interquartile range, with most of the data clustered around the median, that indicates minimal data dispersion within the box plot. This observation persists despite the presence of an outlier. The data exhibit a similar distance range to that of the rodent subclade *Mobatvirus* II intergroup (see Supplementary Fig. S2).

When comparing each formed clade in relation to the others, *Mobatvirus* demonstrates low intergroup dispersion, indicating a small distance. In intragroup analysis, the values had a larger dispersion and were entirely asymmetric. These data imply that *Buritiense* could potentially belong to a new species within bat-borne HVs.

## Discussion

A significant objective of infectious disease research is to understand and characterize the unidentified pathogens present in reservoirs before they manifest in humans. Bats are involved in the transmission cycle of various emerging and re-emerging diseases^6^. Brazil has become a recurrent location for research due to the vast variety of bat species across its different biomes^15^. All known human pathogenic HV are rodent-borne and belong to the *Orthohantavirus*. The HCPS presents a significant public health concern in Brazil, given the numerous reported cases. Infections are often linked to precarious agricultural conditions that foster rodent infestations, as well as inadequate housing in rural areas, rendering individuals susceptible to rodent excretions^16^. Furthermore, ecosystem alterations, such as deforestation, contribute to an imbalance in the proximity dynamics with humans. In urban settings, the virus can also proliferate, as reservoirs thrive in informal urban settlements, easily drawn by food sources and inadequate hygiene practices^17,18^.

Bats also begin to have a different interaction with humans, potentially allowing the virus to encounter permissive cells for replication and the ability to disseminate it.

A study conducted with *Phyllostomidae*, *Molossidae* and *Vespertilionidae* bats captured in the southeastern region of Brazil, between 2012 and 2014, detected IgG antibodies for *Araraquara hantavirus* (ARAQV) using an indirect enzyme-linked immunosorbent assay (ELISA). ARAQV is a pathogenic HV genotype, inserted within the *Andes orthohantavirus* genus, which causes diseases in humans^19^. This supports the idea that these bats become infected with the HV at some point in their lifetime. In another report, HV was detected in *Carollia perspicillata* and *Desmodus rotundus*, both from the *Phyllostomidae* family, by indirect ELISA using a recombinant N protein of ARAQV as antigen. Furthermore, blood samples and tissue from the kidneys, liver, heart, spleen, and lungs were subjected to RT-PCR, confirming the presence of HV in *D. rotundus*. Immunohistochemical analysis revealed positive brown immune-staining for the HV nucleocapsid in hepatocyte cells and striated cardiac muscle cells^7^.

Most HV cases occur in the southern and southeastern regions of Brazil^17^, and due to their higher vulnerability, virus monitoring studies are more frequent in these areas^7–9,19^. However, all reports of HV in bats in Brazil are related to *Orthohantavirus*, here we report a sequence belonging to the *Mobatvirus* genus.

There are studies that discuss the possibility of other mammals could be the source of infection, given the close phylogenetic relationships among certain HV taxa across large geographic areas, supporting the occurrence of long-term virus-host co-divergence among the mammalian orders *Rodentia*, *Chiroptera*, and *Soricomorpha*^20^. Sequences related to *Mobatvirus* genus, including *Xuan son virus*, *Laibin virus*, *Drakong virus* and *Quenzon virus*, that have complete genomes, are described in frugivorous and insectivororus bats from Asia^21^, and some in bats from Africa^22,23^. The limited reports in South America highlight the need for further in-depth studies on the virome of *Chiroptera* fauna in this region.

At Maranhão, only human serologic evidence has indicated the circulation of HV in six municipalities. Notably, no cases of HCPS were officially declared in any of these areas. These findings imply that either mild or asymptomatic cases were taking place, but were not recognized as HCPS, or that cases of HV infection were not being reported. In the state of Ceará, also situated in the Northeastern of Brazil, blood samples were collected from patients suspected of *Dengue virus* infection and subjected to ELISA, using the recombinant N protein of ARAQV as antigen, three tested positive. One presented IgM antibodies HV, while the other two exhibited IgG antibodies to HV^24^. This emphasizes the importance of virological surveillance in other regions, where HV may also circulate in bats, suggesting that moderate or asymptomatic cases may be occurring in these locations.

In the North and Northeastern region of Brazil, there are many rural areas characterized by abundant native plantations used for extractive activities and the local population frequents these areas to derive their livelihood from nature^25,26^. This interaction places them in direct proximity to bats, potentially facilitating viral transmission if these animals are carriers of pathogenic HV.

It is known that frugivorous bats are responsible for the transmission of many RNA viruses^2,6^. In Brazil, a variety of bat species primarily act as reservoirs for the *Rabies virus*. Reports of HV in bats are recent, and it is unknown whether the animals develop the disease or not^27^. Therefore, the feeding dynamics may be related to the viral source, frugivorous bats, such as *Carollia perspicillata*, may share the same food sources with other wildlife, enabling viral transmission between them.

Molecular biology techniques have led to the identification of numerous bat-borne viruses across the globe. Nevertheless, NGS methods have significantly expedited the discovery of novel bat viruses^2^. In the analysis of bat tissue from East Africa, a novel virus named *Kiwira virus* was identified within the genus *Mobatvirus*. This discovery revealed a closer phylogenetic proximity to the *Quezon virus* and *Robina virus*^23^. Just as in our analyses, it’s also observed the formation of a branch close to *Robinaense* and *Quezonense* viruses with *Buritiense* in the phylogenetic tree.

The recent discovery was *Brno virus* in the European bats collected in the Czech Republic during 2008–2013, sequence comparison revealed that this virus showed 54.7–78.3% nt and 44.5–81.7% aa sequence identity with other bat-borne HV. Interestingly, it clustered more closely with the *Longquan virus*, found in Chinese bats^20,28^. In our findings, the obtained sequences showed a closer affinity with HVs from Southeast Asian bats.

Nevertheless, the genomes of bat-borne hantaviruses are generally described as incomplete. The limited detection may be attributed to the high divergence of their genomes, as well as the localized nature of infection, small sample sizes, primer incompatibilities, suboptimal PCR cycling conditions, and variable tissue preservation with degraded RNA^21,29^. The study sequence is part of a viral metagenomic analysis for surveillance purposes, making it crucial to investigate whether there are sequences related to viral families that cause diseases in humans. Therefore, even though we have not been able to obtain the complete sequence of this HV, it is a significant discovery indicating the presence of *Mobatvirus* circulating in Brazilian bats.

## Methods

### Site and sample collection

A metagenomic study was conducted for the virological surveillance of RNA hosted in bats inhabiting specific areas in the state of Maranhão, located in Northeastern Brazil. The capture was authorized by the Ethics Committee on Animal Experimentation of State University of Maranhão, number 025/2019 and adhered to the prescribed research protocols in accordance with the ARRIVE guidelines^30^. All methods were conducted in accordance with the guidelines and regulations outlined in the American Veterinary Medical Association (AVMA) Guidelines for the Euthanasia of Animals (2020).

Three frugivorous bats, *Carollia perspicillata*, were captured using mist nets in a district of Timon City, Maranhão, named Buriti Cortado (5^∘^ 09’ 21.5” S 43^∘^ 11’ 33.4” W) in October 2021 Fig. 3. They were anesthetized with ketamine/xylazine and euthanized by exsanguination. The tissues (liver, lung, heart, and kidney) were collected and stored in liquid nitrogen until transportation to the Institute, where they were stored at $-80^\circ \hbox {C}$. The samples were grouped in three tubes for analysis.

**Figure 3:**
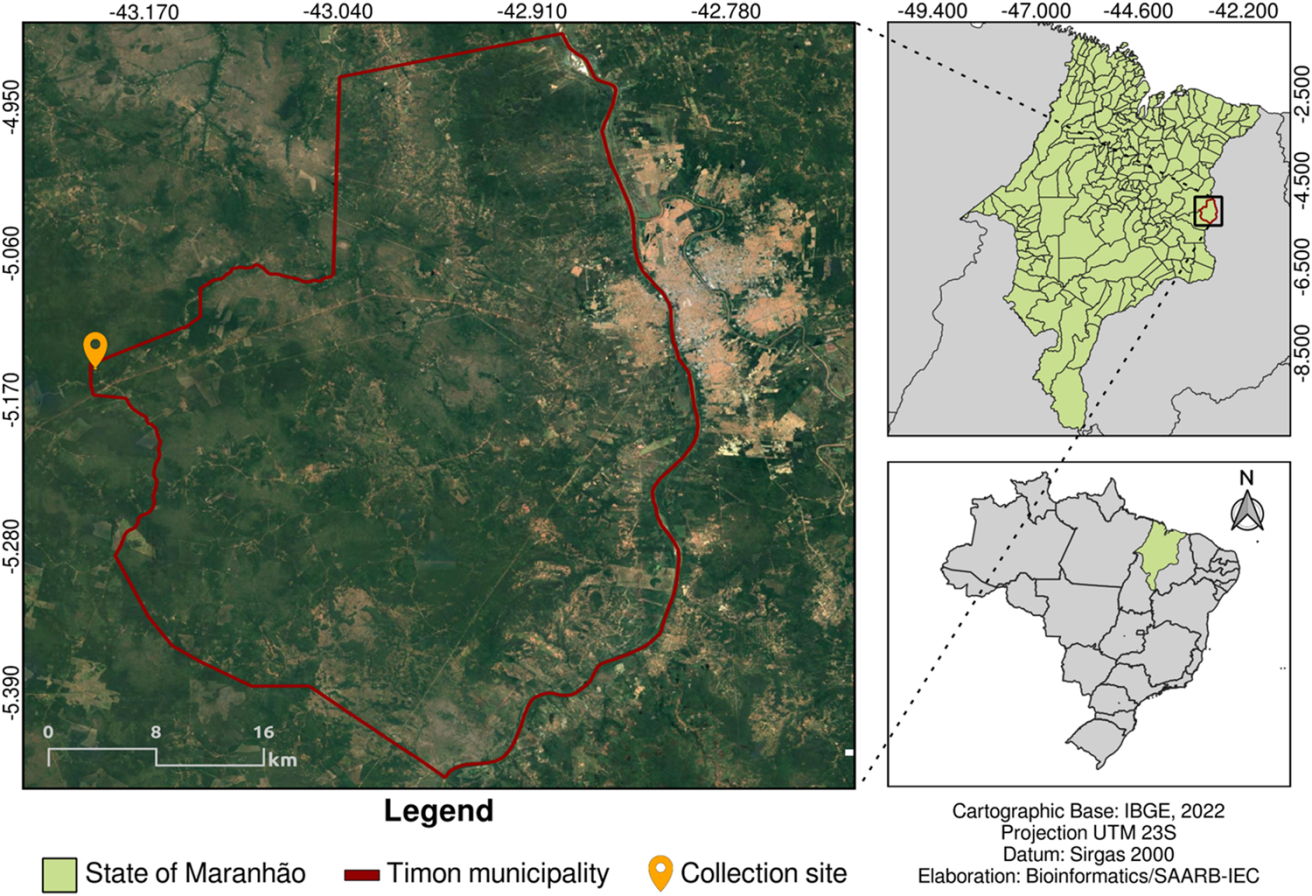
Map of the collection site in Timon, Maranhão The region is situated in the Northeastern of Brazil. The figure was generated using QGIS v.3.10 software (available at https://qgis.org/en/site/), which utilized satellite approximations from the Google Maps application in conjunction with the IBGE 2022 cartographic database (available at https://www.ibge.gov.br/).

### RNA extraction, cDNA synthesis and sequencing

In this study, a pool of heart, lung, and kidney was used in a unique tube for the isolation of total RNA. The process was initiated using the TRIzol^TM^ Plus RNA Purification Kit and a 5 mm tungsten bead using the TissueLyser II system (Qiagen, Hilden, Germany) for 2 min at 25 Hz, adhering to the prescribed protocols provided by the manufacturer. Following this step, the concentration of RNA was quantified employing the Qubit^®^ RNA HS Assay Kit in conjunction with the Qubit^®^ 2.0 Fluorometer (Invitrogen), while RNA integrity was assessed through the application of the Agilent RNA 6000 Pico Kit on the Bioanalyzer 2100 system (Agilent Technologies).

The synthesis of complementary DNA (cDNA) was carried out, utilizing the SuperScript^TM^ IV VILO^TM^ Master Mix (Invitrogen) for the initial strand synthesis, and the Second Strand cDNA Synthesis Kit (Invitrogen) for the synthesis of the second strand. The resultant cDNA was subsequently subjected to a purification process using the PureLink^TM^ PCR Purification Kit (Invitrogen).

The generation of sequencing libraries was facilitated by the Nextera XT DNA Library Preparation Kit designed for Illumina^®^ platforms, meticulously following the manufacturer’s recommended procedures. Accurate quantification of the library was performed employing the Qubit^®^ dsDNA HS Assay kit, while fragment sizes were precisely evaluated with the High Sensitivity DNA Analysis Kit on the Bioanalyzer 2100 (Agilent Technologies).

High-throughput sequencing was conducted on an Illumina^®^ NextSeq 500 platform, employing the NextSeq 500/550 High Output v2.5 (300 cycles) kit, thus generating paired-end sequencing reads for comprehensive data analysis.

### Bioinformatic analysis

Fastp was employed to assess the quality of raw reads and eliminate short reads, those with low quality, and undetermined bases^31^. The generated results were assembled using the *De Novo* method in the MEGAHIT v1.2.9 assembler^32^ and aligned against non-redundant protein and nucleotide databases using DIAMOND^33^ and KRAKEN2^34^ respectively, with a significance threshold set at $10^{-5}$.

Contigs were further examined using MEGAN6, KronaTools v2.8.1, and the Pavian v1.0 software, which provided taxonomic classifications corresponding to the viral contigs^35–37^. Subsequently, the contig was mapped against the *raw data* and aligned to a reference sequence using Geneious v.9.1.8^38^.

### Identity/divergence analysis

The partial genome obtained was compared with 37 RefSeq genomes from the subfamily *Mammahantavirinae* and an outgroup from the subfamily *Repantavirinae*. All sequences are available in Table S2. The multiple alignment of the dataset and the sequences of this study were performed using Clustal W for nucleotide and amino acid analysis^39^. The partial genome sequence of HV was deposited in GenBank under the accession number OR684449.

The identity and divergence matrices were generated using Geneious v.9.1.8 and MEGA X^40^. The amino acid identity matrix, generated by MEGA X using the amino acid substitution p-distances, was converted, using the R programming language, into a boxplot graphs illustrating intragroup and intergroup distances/divergence between members of the same genus when compared to each other and in relation to new sequence of HV.

### Phylogenetic analysis

Phylogenetic inference was based on the amino acid sequences of various virus strains belonging to the *Hantaviridae* family, available in the National Center for Biotechnology Information database (http://www.ncbi.nlm.nih.gov). The coding regions of the L segment were utilized for analysis.The set of sequences, including the sample from the present study, underwent Multiple Sequence Alignment (MSA), followed by manual inspection using Geneious v.9.1.8 software (available at https://www.geneious.com/).

Phylogenetic inferences were conducted using the *Maximum Likelihood* (ML) method, implemented in the IQ-TREE v.2^41^, employing the LG+F+R5 substitution model and employing 1000 bootstrap replicates for statistical support^42^. The visualization and editing of the resulting trees were conducted using FigTree (available at http://tree.bio.ed.ac.uk/software/figtree/) and Inkscape (https://inkscape.org/), respectively.

### Ethical approval and informed consent

The project was authorized by the Ethics Committee on Animal Experimentation of State University of Maranhão, number 025/2019 and adhered to the prescribed research protocols in accordance with the ARRIVE guidelines.

## Conclusion

The report of HV in bats from Northeastern Brazil is intriguing. Further analysis regarding the viral load in different tissues and viral isolation, followed by infection experiments, are necessary to provide additional insights and confirm whether bats indeed act as reservoirs. This study raises concerns about its potential transmission to the human population. Understanding the viral diversity carried by these species can aid in staying alert about potential spillover occurrences.

### Supplementary Information

Supplementary Figure S1.

Supplementary Figure S2.

Supplementary Tables.

## Data Availability

The consensus sequence of this research have been submitted to the National Center for Biotechnology Information under accession OR684449 and it’s available at December 13/2023.
