# Supplementary figures and images for: A novel hantavirus identified in bats (Carollia perspicillata) in Brazil
Source: Sci Rep. 2024 Mar 15;14:6346. doi: 10.1038/s41598-024-56808-6 (PMC10943075; doi:10.1038/s41598-024-56808-6)

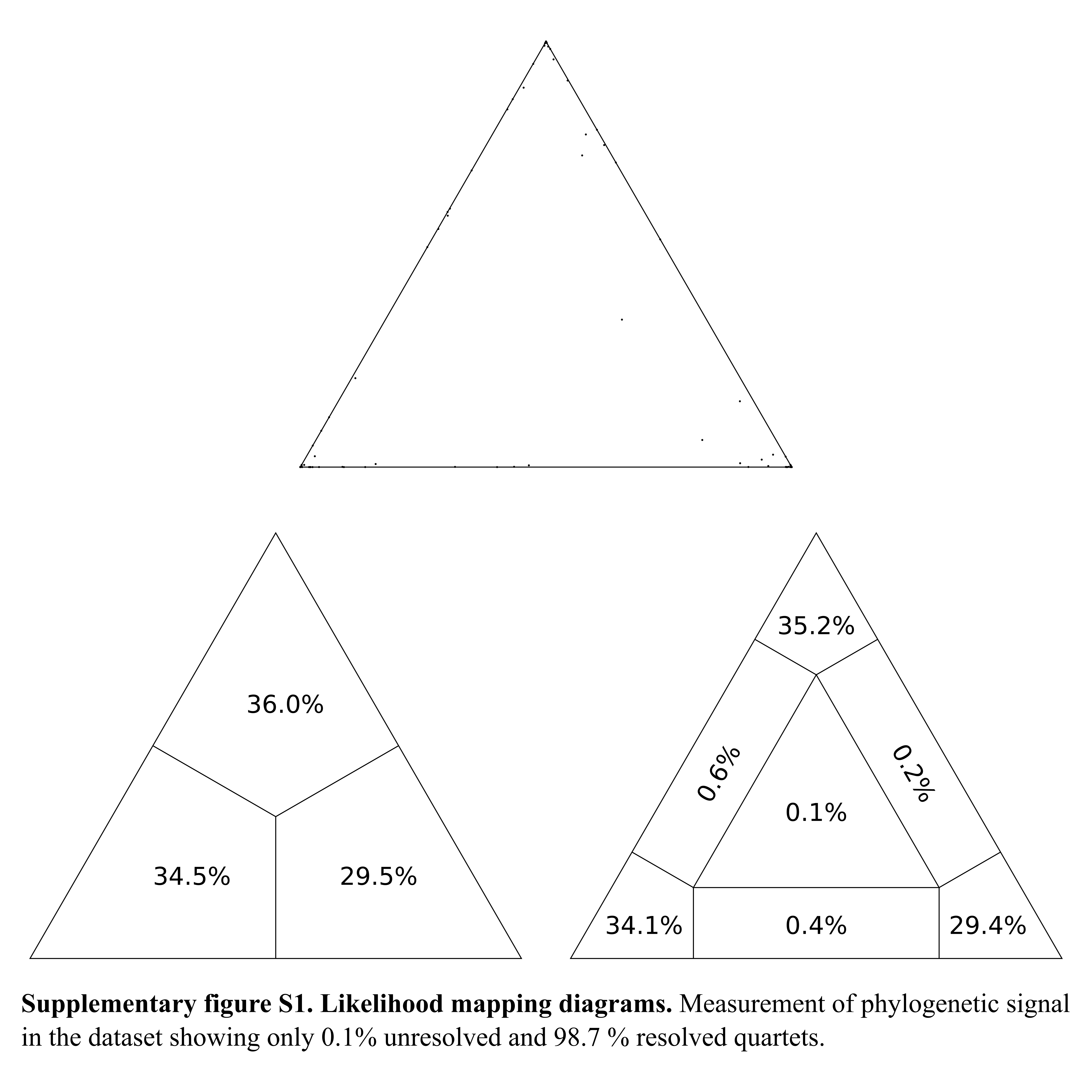

Supplement: Supplementary file 1 — Supplementary Figure S1. [file 41598_2024_56808_MOESM1_ESM.jpg]
